# Supplementary material for: Size and Flexibility Define the Inhibition of the H3N2 Influenza Endonuclease Enzyme by Calix[n]arenes
Source: Antibiotics (Basel). 2019 Jun 3;8(2):73. doi: 10.3390/antibiotics8020073 (PMC6627454; doi:10.3390/antibiotics8020073)
Supplement: Supplementary file 1 [file antibiotics-08-00073-s001.pdf]

Supplementary Information

Size and Flexibility Define the Inhibition of the H3N2 Influenza Endonuclease Enzyme by Calix[n]arenes

Yannick Tauran [1,2,*], Jose Pedro Cerón-Carrasco [3], Moez Rhimi [4], Florent Perret [5], Beomjoon Kim [2,6], Dominique Collard [7], Anthony W. Coleman [1] and Horacio Pérez-Sánchez [3,*]

1 LMI CNRS UMR 5615, Université Lyon 1, Villeurbanne, 69622, France; anthony.coleman@univ-lyon1.fr
2 LIMMS/CNRS-IIS UMI 2820, Institute of Industrial Science, The University of Tokyo, Tokyo, 153-8505, Japan; bjoonkim@iis.u-tokyo.ac.jp
3 Bioinformatics and High Performance Computing (BIO-HPC) Research Group, Universidad Católica de Murcia (UCAM), Murcia 30107, Spain; hperez@ucam.edu
4 INRA, UMR 1319 Micalis, F-78350 Jouy-en-Josas, France; moez.rhimi@jouy.inra.fr
5 ICBMS, CNRS UMR, Université Lyon 1, Villeurbanne, 69622, France; florent.perret@univ-lyon1.fr
6 CIRMM, Institute of Industrial Science, University of Tokyo, Tokyo, Japan
7 Univ. Lille, CNRS, Centrale Lille, ISEN, Univ. Valenciennes, UMR 8520 - IEMN, Lille, F59000, France; collard@iis.u-tokyo.ac.jp

* Correspondence: yannick.tauran@univ-lyon1.fr (Y.T.); hperez@ucam.edu (H.P.-S.); Tel: +81-3-5841-7073 (Y.T.); Tel.: +34-968-278819 (H.P.-S).

Supplementary Materials:

Figure S1: Coomasie-stained gel SDS-PAGE of the PA protein purified from bacterial extract. Molecular weight (MW) ladder (kDa) is shown on the right.

Figure S2: Agarose gel electrophoresis shows the digestion activity of PA endonuclease protein. A concentration range of PA endonuclease protein has been mixed with λ-DNA phage. Molecular weight (MW) ladder (1kb) is shown on the right. Reaction products were run in a 0.6 % agarose gel stained with Ethidium Bromide.

Figure S3. IC50 determination of H3N2 influenza PA endonuclease at 6.4 μM of SC8a on PA endonuclease. '+' means DNA and '-' corresponds to DNA and PA endonuclease.

Figure S4. IC50 determination of H3N2 influenza PA endonuclease at 14 μM of SC8b on PA endonuclease. '+' means DNA and '-' corresponds to DNA and PA endonuclease.

Figure S5. IC50 determination of H3N2 influenza PA endonuclease at 18.7 μM of SC8c on PA endonuclease. '+' means DNA and '-' corresponds to DNA and PA endonuclease.

Figure S6. IC50 determination of H3N2 influenza PA endonuclease at 11.2 μM of SC6a on PA endonuclease. '+' means DNA and '-' corresponds to DNA and PA endonuclease.

Figure S7: Histogram obtained from blind docking results. Estimated binding energy in Kcal/mol is shown on X-axis, while the number of poses or occurrences with binding energy values corresponding to a given interval is shown on Y axis.

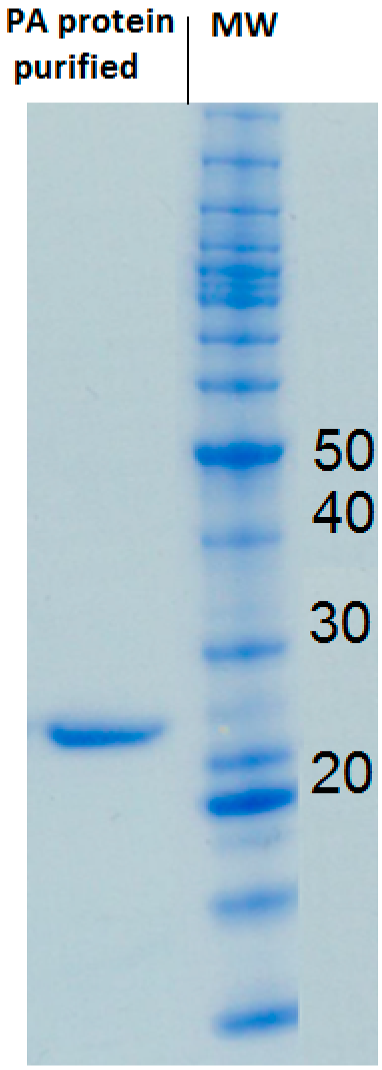

Figure S1. Coomasie-stained gel SDS-PAGE of the PA protein purified from bacterial extract. Molecular weight (MW) ladder (kDa) is shown on the right.

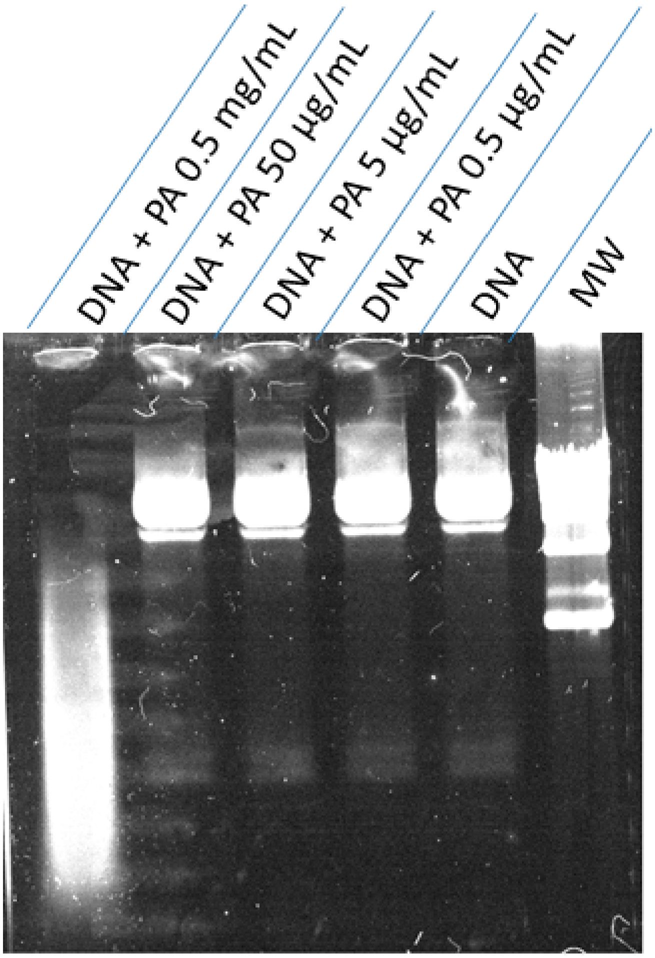

Figure S2. Agarose gel electrophoresis shows the digestion activity of PA endonuclease protein. A concentration range of PA endonuclease protein has been mixed with λ-DNA phage. Molecular weight (MW) ladder (1kb) is shown on the right. Reaction products were run in a 0.6 % agarose gel stained with Ethidium Bromide.

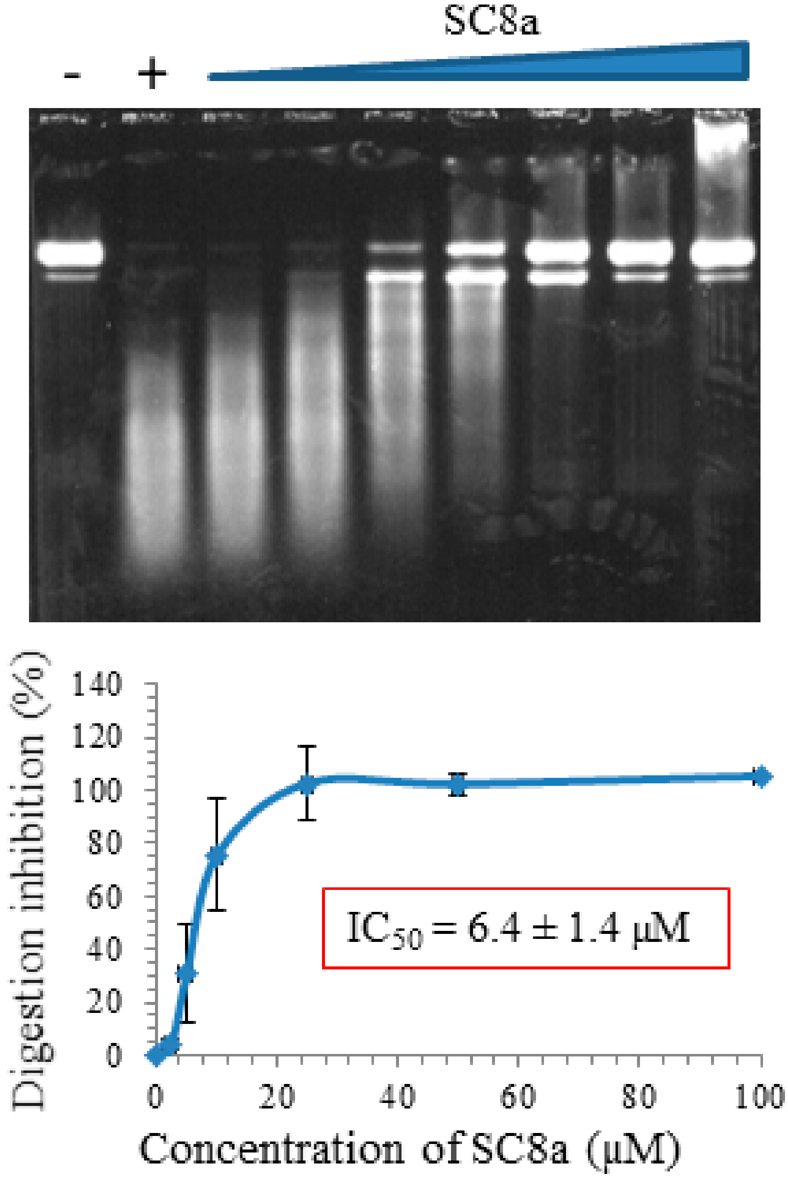

Figure S3. IC50 determination of H3N2 influenza PA endonuclease at 6.4 μM of SC8a on PA endonuclease. '+' means DNA and '-' corresponds to DNA and PA endonuclease.

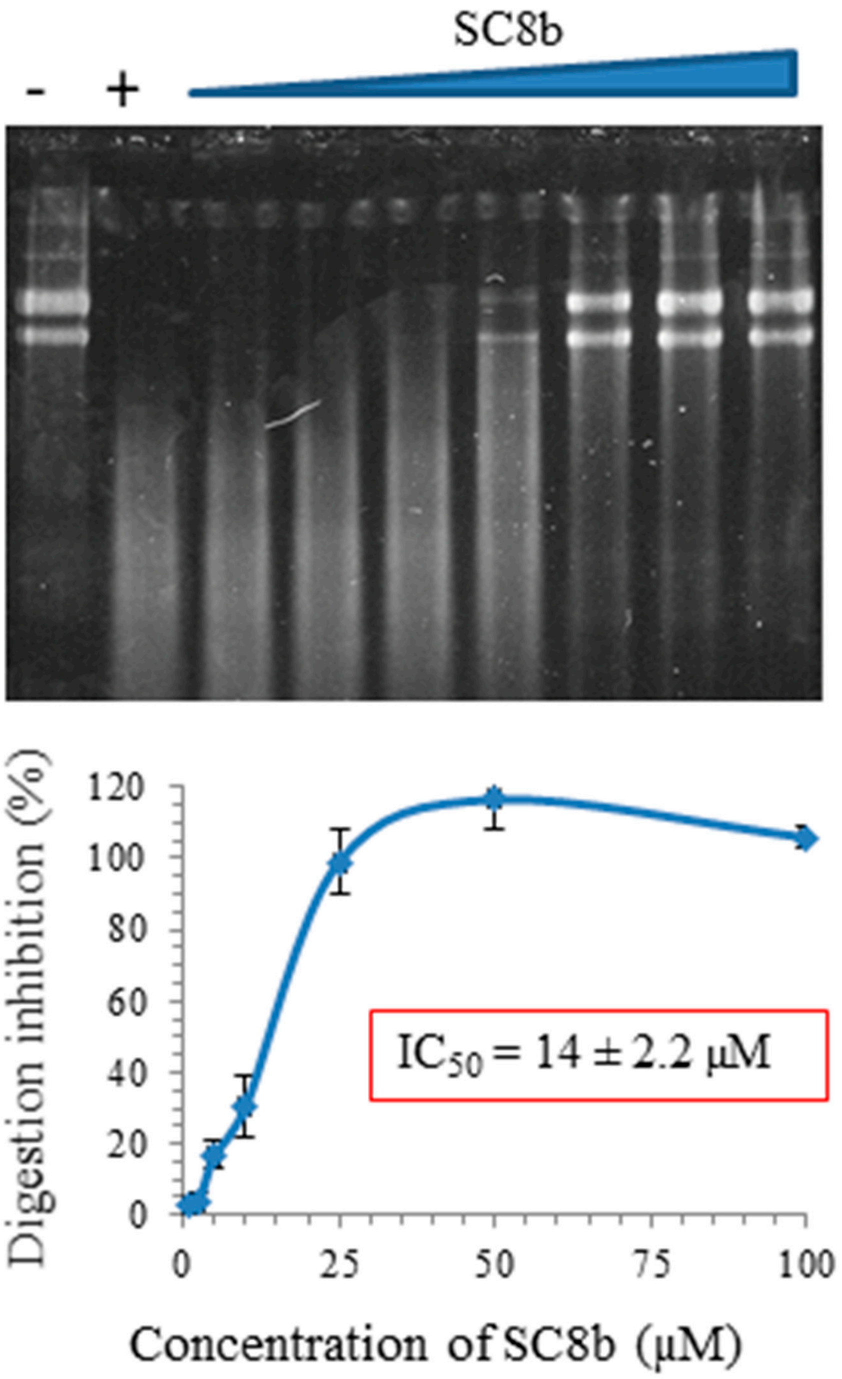

Figure S4. IC50 determination of H3N2 influenza PA endonuclease at 14 μM of SC8b on PA endonuclease. '+' means DNA and '-' corresponds to DNA and PA endonuclease.

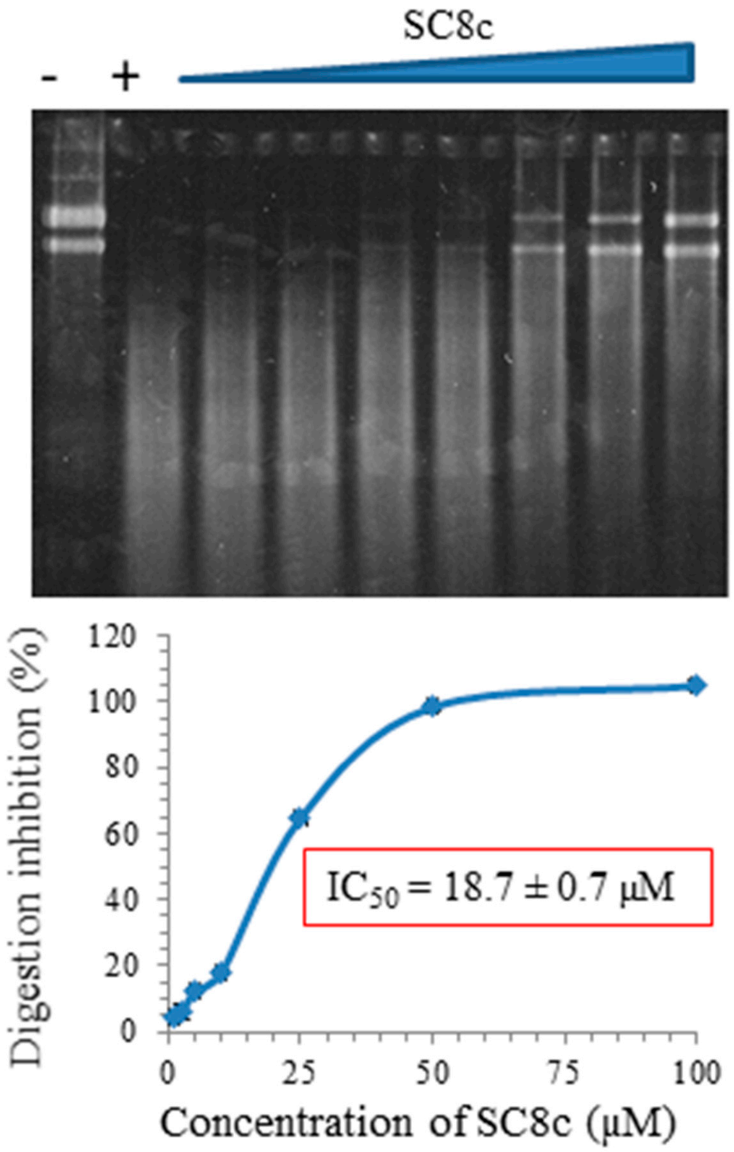

Figure S5. IC50 determination of H3N2 influenza PA endonuclease at 18.7 μM of SC8c on PA endonuclease. '+' means DNA and '-' corresponds to DNA and PA endonuclease.

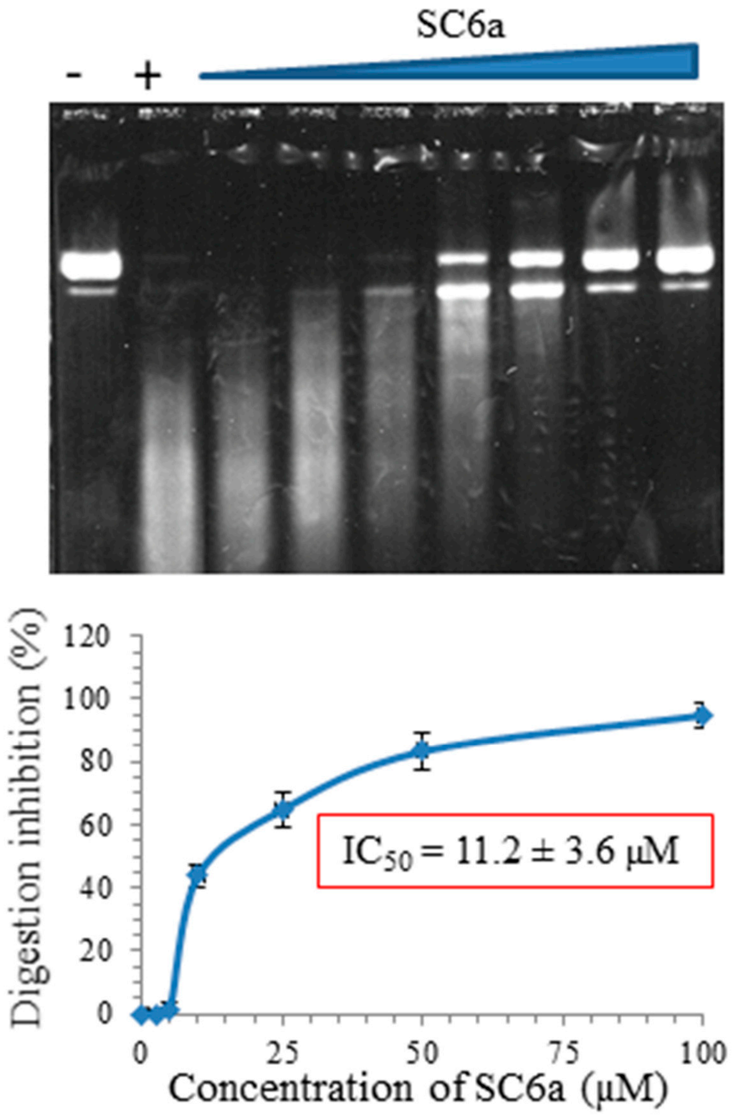

Figure S6. IC50 determination of H3N2 influenza PA endonuclease at 11.2 µM of SC6a on PA endonuclease. '+' means DNA and '-' corresponds to DNA and PA endonuclease.

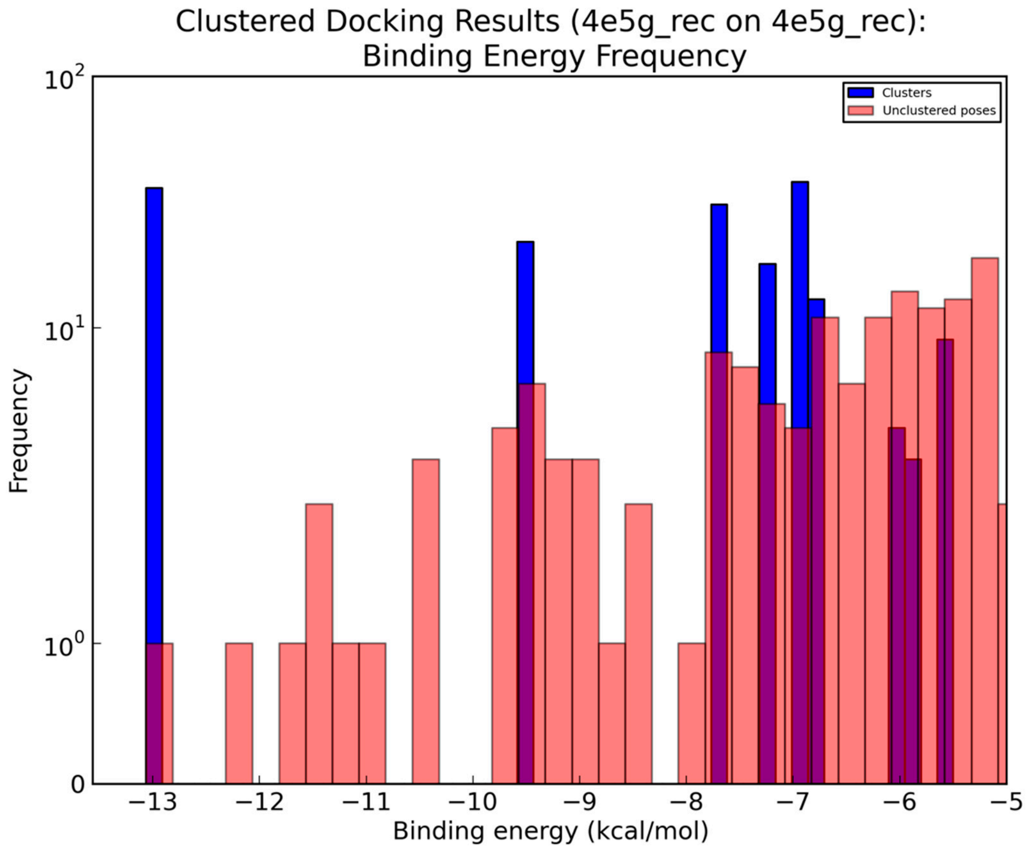

Figure S7. Histogram obtained from blind docking results. Estimated binding energy in Kcal/mol is shown on X-axis, while the number of poses or occurrences with binding energy values corresponding to a given interval is shown on Y axis.

© 2019 by the authors. Submitted for possible open access publication under the terms and conditions of the Creative Commons Attribution (CC BY) license (http://creativecommons.org/licenses/by/4.0/).
